# 1-Deoxynojirimycin Combined with Theaflavins Targets PTGS2/MMP9 to Exert a Synergistic Hypoglycemic Effect

**DOI:** 10.3390/nu18010099

**Published:** 2025-12-27

**Authors:** Yuanyuan Wang, Chenyin Qu, Qiannan Di, Jingyi Zhang, Lixin Na

**Affiliations:** The College of Public Health, Shanghai University of Medicine & Health Sciences, 279 Zhouzhu Road, Pudong New Area, Shanghai 201318, China; wangyy@sumhs.edu.cn (Y.W.); qcy1008@126.com (C.Q.); diqn@sumhs.edu.cn (Q.D.); zhangjy@sumhs.edu.cn (J.Z.)

**Keywords:** 1-deoxynojirimycin, theaflavins, type 2 diabetes mellitus (T2DM), synergistic

## Abstract

**Background**: This study aimed to explore the synergistic hypoglycemic effect and mechanism of 1-deoxynojirimycin (DNJ) in mulberry leaves and theaflavins (TFs) in black tea. **Methods**: The synergistic inhibition of α-glucosidase and α-amylase by DNJ-TFs was evaluated using enzyme assays and the Chou–Talalay model. Insulin-resistant (IR) HepG2 cells and high-fat diet (HFD)-induced type 2 diabetes mellitus mice were treated with DNJ, TFs, or DNJ-TFs, determining the efficacy of drug combinations by measuring glycolipids and inflammatory factors. Network pharmacology and molecular docking were used to identify key target genes and signaling pathways, and CETSA experiments were used to verify the binding of drugs to targets. Key genes were further verified by immunofluorescence, Western blot, and Real-time PCR. **Results**: DNJ-TFs synergistically suppressed α-glucosidase (CI = 0.85) and α-amylase (CI = 0.76). In HepG2 cells, DNJ-TFs ameliorated palmitic acid-induced IR by promoting glucose uptake, attenuating lipid accumulation, and regulating glycolipid metabolism. In HFD mice, DNJ-TFs counteracted hyperglycemia, dyslipidemia, systemic inflammation and oxidative stress, elevated HOMA-IR, and hepatic steatosis. Network pharmacology integrated with experimental validation identified PTGS2 and MMP9 as key binding targets of DNJ and TFs. Furthermore, DNJ-TFs could inhibit the increase in liver TNFα protein and the decrease in *p*-AKT, *p*-GSKα, *p*-GSKβ, and GLUT2 protein caused by high fat, both in vivo and in vitro. **Conclusions**: DNJ and TFs exert synergistic glucose-lowering effects by targeting PTGS2/MMP9 and regulating the TNFα/AKT/GSK3/GLUT2 axis, providing a promising natural therapeutic strategy for diabetes management.

## 1. Introduction

According to the International Diabetes Federation (IDF) report in 2021, a total of 537 million people live with diabetes mellitus globally, and it is estimated that this number will rise to 643 million by 2030 and 783 million by 2045 [1]. As an important public health issue, patients with type 2 diabetes mellitus (T2DM) are usually accompanied by a range of severe complications, including diabetic foot conditions, retinopathy, nephropathy, neuropathy, and macrovascular diseases [2,3]. Currently, the treatment of T2DM mainly comprises using drugs, such as metformin, acarbose, and insulin injections [4]. However, long-term use of these drugs can lead to serious side effects [5]. In recent years, researchers have discovered that functional components in plants have great potential in preventing or alleviating chronic diseases [6].

Traditional Chinese medicine theory believes that mulberry leaves are cold in nature, belong to the lung and liver meridians, and have the effect of dispersing wind-heat, thus clearing the lungs and moistening dryness. Black tea is warm in nature and can warm the stomach and dispel cold. The combination of the two can neutralize cold and heat, while preventing mulberry leaves from being too cold and damaging the stomach. 1-deoxynojirimycin (DNJ) is a representative alkaloid in Morus plants. Discovered as an effective antidiabetic α-glucosidase inhibitor, in traditional Chinese medicine, these two ingredients are often used together to treat diabetes. DNJ has gradually exhibited excellent biofunctions involved in lipid metabolism, maintenance of metabolic homeostasis, and amelioration of diabetic complications [7]. Theaflavins (TFs) are the main chemical components of black tea and have been reported to have antioxidant, anticancer, anti-inflammatory, antibacterial, and antiviral properties [8,9]. In addition, TFs are lipophilic and have anti-obesity and anti-hypertriglyceridemia effects, which have great potential in preventing and improving diabetes, metabolic syndrome, and other complications [10,11,12]. Current research largely focuses on the individual effects of DNJ and TFs, and their mechanisms of action remain unclear. Furthermore, whether DNJ and TFs can exert a synergistic effect in improving type 2 diabetes has not yet been explored.

Driven by progress in high-throughput sequencing and computer science, huge new bioinformatics networks have emerged. Network pharmacology, one such bioinformatics network, aims to construct a multilevel network through various database searches, high-throughput omics data analysis, and computer simulations to analyze the relationship of medicines, diseases, and targets [13]. Therefore, this study explores the mechanism of synergistic hypoglycemia of DNJ combined with TFs by combining network pharmacology and biological experiments, and in vivo and in vitro experiments, so as to provide a sufficient scientific basis for the development of related hypoglycemic products and the prevention and control of the development of T2DM.

## 2. Materials and Methods

### 2.1. α-Glucosidase and α-Amylase Enzyme Inhibition Assay and Chou–Talalay Model

DNJ (Macklin, Shanghai, China) and TFs (Titan Scientific, Shanghai, China) were prepared into test solutions with concentrations of 30, 20, 10, 5, and 1 μg/mL, respectively. At the same time, DNJ and TFs solutions of the same concentration were selected and mixed in equal volumes to detection using an α-glucosidase assay kit (Sangon Biotech, Shanghai, China) and an α-amylase assay kit (Solarbio, Beijing, China) in vitro. Meanwhile, in order to find the optimal ratio of DNJ and TFs for synergistic hypoglycemic effect, we selected the final concentrations of DNJ and TFs as 5, 10, and 20 μg/mL, mixed DNJ and TFs in ratios of 1:1, 1:2, 1:3, 3:1, and 2:1, and conducted α-glucosidase inhibition experiments.

The α-glucosidase inhibition assay was performed as described by Franco et al. [14]. Briefly, a mixture containing 50 μL of phosphate-buffered saline (PBS; 100 mmol/L, pH 6.8), 20 μL of α-glucosidase solution (0.5 U/mL), and 10 μL of the sample solution was prepared in a 96-well plate. The mixture was pre-incubated at 37 °C for 5 min, after which the reaction was initiated by adding 20 μL of the substrate *p*-nitrophenyl-α-d-glucopyranoside (5 mmol/L). Following a 15 min incubation at 37 °C, the reaction was terminated with 50 μL of sodium carbonate solution (0.2 mol/L). After standing for 15 min, the absorbance was measured at 405 nm.

The α-amylase inhibition assay was carried out according to the procedure reported by Podsedek et al. [15] with several modifications. In a centrifugal tube, the sample (50 μL), α-amylase solution (50 μL), and phosphate-buffered solution (100 μL, pH 6.8) were mixed, to which 1% soluble starch (100 μL) was added, followed by reaction at 37 °C for 10 min. Then, 3,5-dinitrosalicylic acid (200 μL) was used to color the solution for 5 min in a 100 °C water bath, and the mixture was finally diluted to 4 mL with distilled water to detect the absorbance at 405 nm.

The Two-way ANOVA and Chou–Talalay combination index method were used to analyze the interaction of the two components. Compusyn software version 1.0 was used to perform drug synergy studies and calculate the measurement combination index (CI) to determine the interaction between the two components. When CI > 1, the two were antagonistic; when CI = 1, the two were additive; when CI < 1, the two were synergistic [16].

### 2.2. Cell Culture and Treatment

HepG2 cells were obtained from the Cell Bank of the Chinese Academy of Sciences (Shanghai, China). Cells were cultured in Dulbecco’s modified Eagle medium (DMEM) (HyClone, South Logan, UT, USA) supplemented with 10% fetal bovine serum (PAA Laboratories, Pasching, Austria), and 100 IU/mL penicillin and 100 μg/mL streptomycin (Beyotime, Shanghai, China). The cells were cultured in a 5% CO_2_ incubator at 37 °C.

Palmitic acid (PA), a major saturated fatty acid, was used to construct a model of high-fat-induced hepatic IR in vitro [17]. The cells were divided into six groups according to different treatment measures: control group, model group (300 μM PA (Beyotime, Shanghai, China)), positive drug (300 μM PA + 10 μg/mL metformin (Titan, Shanghai, China)) group, treatment group A (300 μM PA + 10 μg/mL DNJ + 5 μg/mL TFs), and treatment group B (300 μM PA + 20 μg/mL DNJ + 10 μg/mL TFs). After 24 h of treatment, the cells were collected for subsequent experiments.

In this experiment, MMP9 siRNA and PTGS2 overexpression plasmid (RiboBio, Guangzhou, China) were used. Specifically, HepG2 cells were transfected with 1 μg/mL PTGS2 overexpression plasmid or 100 nM MMP9 gene siRNA using Lipofectamine 2000 reagent (Invitrogen, Carlsbad, CA, USA) and detected after 48 h according to the manufacturer’s instructions.

### 2.3. Cell Viability Assay

Cell viability was detected by Cell Counting Kit-8 (CCK-8) assay (Sangon Biotech, Shanghai, China). A total of 5 × 10^3^ cells/well were seeded into 96-well plates and incubated for 24 h. The cells were treated with DNJ (50, 40, 30, 20, 10 μg/mL), TFs (40, 30, 20, 10, 5 μg/mL) and PA (0, 100, 200, 300, 400 μM) for 24 h, with 3 replicates in each group, and then cultured in the incubator for another 24 h. Then, 10 μL of CCK-8 was added to each well, and the samples were incubated for 2 h. Finally, the samples were evaluated according to the optical density (OD) measured at 450 nm using a ReadMax 1500 Absorption Full Wavelength Microplate Reader (Shanpu, Shanghai, China).

### 2.4. Cellular Glucose Uptake, Glucose Consumption, and Glycogen Content Assays

Cellular glucose uptake was measured using the fluorescent glucose analog 2-NBDG (Invitrogen, CA, USA). Briefly, after washing with PBS, HepG2 cells with different treatments were placed in glucose-free medium (Solarbio, Beijing, China), incubated with 100 nM insulin (Sigma, St. Louis, MO, USA) for 15 min, and then incubated with 0.1 mM 2-NBDG at 37 °C for 30 min. The fluorescence intensity of cells in each group was detected using the instructions of the glucose detection kit (Solarbio, Beijing, China) or flow cytometry.

Glucose consumption was calculated by detecting the glucose content in the culture medium before and after the different treatments using a Glucose Assay kit (Solarbio, Beijing, China). Cellular glycogen content was determined using the Glycogen Content Assay kit (Solarbio, Beijing, China) according to the manufacturer’s protocol. All experiments were conducted with a minimum of three replicates, and the resulting data were normalized to the total protein content.

### 2.5. IL-1β, TNF-α, IL-6, Superoxide Dismutase (SOD), Glutathione (GSH), and Malondialdehyde (MDA) Levels and Anti-COX Effects

According to the operating instructions, the cells and animal serums were tested using the IL-1β detection kit (Beyotime, Shanghai, China), TNF-α detection kit (Beyotime, Shanghai, China), IL-6 detection kit (Beyotime, Shanghai, China), and COX-2 inhibitor screening kit (Beyotime, Shanghai, China).

### 2.6. Lipid Content Measurement

For Oil Red O staining, HepG2 cells were rinsed three times with PBS and then incubated with 1 μg/mL Oil Red O staining solution (Invitrogen, Carlsbad, CA, USA) for 20 min. Then, cells were rinsed three times with PBS and photographed with an inverted microscope and saved.

Triglycerides (TG) and Total cholesterol (TC) contents in cells and animal sera were measured using a TG kit (Solarbio, Beijing, China) and a TC kit (Solarbio, Beijing, China) according to the manufacturer’s instructions. All experimental data were normalized by cell protein content or tissue mass.

### 2.7. Animal Experiments

A total of 56 healthy, 4-week-old male C57BL/6J mice (Cavens, Jiangsu, China) were housed in cages under standard laboratory conditions (at a temperature of 22–24 °C, 50–70% relative humidity, and a 12 h/12 h light/dark cycle). After one week of acclimatization feeding, 56 mice were randomly divided into 7 groups and kept in single cages. Of these, 8 mice in the blank control group (control group) continued to be fed SPF-grade basal diet (14.4% fat, 60.0% carbohydrates, 26.6% protein, Slecas, Shanghai, China.); 8 mice in the model group (model group) were fed SPF-grade high-fat diet (HFD) (60% fat, 20.0% carbohydrate, 20.0% protein, Ruixin Biotechnology, Shanghai, China); 8 mice in the acarbose positive control group (acarbose group) were fed SPF-grade HFD, and were also orally administered 50 mg/kg of acarbose daily. Furthermore, 8 mice in the DNJ group were fed SPF-grade HFD, and were orally administered 200 mg/kg of DNJ daily; 8 mice in the TFs group were fed SPF-grade HFD, and were orally administered 100 mg/kg of TFs daily; 8 mice in the DNJ-TFs synergistic low-dose administration group (group A) were fed SPF-grade HFD, and were orally administered 100 mg/kg of DNJ and 50 mg/kg of TFs daily; 8 mice in the DNJ-TFs synergistic high-dose administration group (group B) were fed SPF-grade HFD, and were orally administered 200 mg/kg of DNJ and 100 mg/kg of TFs daily. The weight of the mice was recorded weekly. In week 10, after a 12 h fast, blood glucose levels were measured using an Accu-Chek Performa blood glucose meter (Roche Diagnostics GmbH, Mannheim, Germany). A T2DM model was considered successfully established when mice in the model group showed two consecutive blood glucose readings between 11.10 and 16.00 mmol/L, along with symptoms such as polydipsia and polyuria. During the rearing process, animals were immediately euthanized and excluded if they exhibited any of the following: a weight loss of ≥20% from baseline, blood glucose levels consistently ≥ 400 mg/dL for more than 48 h (indicating a risk of diabetic ketoacidosis), inability to obtain food/water, severe lethargy (immobility > 4 h), respiratory distress (rapid breathing > 100 breaths/minute), cyanosis, hypothermia (<32 °C), diarrhea/vomiting lasting more than 24 h, or neurological deficits such as hind limb paralysis.

At the end of the experiment, all mice were subjected to an intraperitoneal glucose tolerance test (IPGTT). After being sacrificed, blood samples and liver tissue were quickly collected for analysis. The study was approved by the Institutional Animal Care Committee from Shanghai University of Medicine and Health Science and conducted following the university guidelines for the care and use of laboratory animals; the approval number is 2022SY011.

### 2.8. IPGTT

IPGTTs were performed as described previously [18]. In brief, the mice were fasted for 12 h and then given glucose by intraperitoneal injection (2 g/kg body weight). Blood samples were obtained at 0, 15, 30, 60, and 120 min, and serum glucose was measured with an Accu-Chek Performa glucometer (Roche Diagnostics GmbH, Mannheim, Germany). The area under the curve (AUC) for glucose tolerance was calculated using the trapezoidal method according to the following formula: AUC = (G0 + G15)/2 × 15 + (G15 + G30)/2 × 15 + (G30 + G60)/2 × 30 + (G60 + G120)/2 × 60. Where G0, G15, G30, G60, and G120 represent blood glucose concentrations (mmol/L) at respective time points, and the coefficients represent time intervals in minutes.

### 2.9. Serum Lipids, Insulin, and Homeostasis Model Assessment (HOMA)-IR

Serum TC, TG, high-density lipoprotein cholesterol (HDL-C), and low-density lipoprotein cholesterol (LDL-C) were examined with assay kits according to the user instructions. Mouse insulin was measured with a rat/mouse insulin ELISA kit (LINCO Research, St Charles, MO, USA). HOMA-IR = fasting glucose (mmol/L) × fasting insulin (mU/L)/22.5.

### 2.10. Hematoxylin–Eosin (HE) Staining

HE staining was conducted according to routine procedures [19]. In brief, after deparaffinization and rehydration, 5 μm longitudinal sections were stained with hematoxylin solution for 5 min, followed by 5 dips in 1% acid ethanol and then rinsed in distilled water. Then, the sections were stained with eosin solution for 3 min and followed by dehydration with graded alcohol and clearing in xylene. The tissue sections were photographed and preserved in a digital scanning microscopic imaging system (3DHISTECH, Budapest, Hungary).

### 2.11. Screening of Potential Targets of DNJ, TFs, and T2DM

The 2D structures of DNJ and TFs were imported into Swiss Target Prediction (http://www.swisstargetprediction.ch/, accessed on 7 November 2021) databases and Pharm Mapper (http://www.lilab-ecust.cn/pharmmapper/, accessed on 7 November 2021) databases, respectively, and Homo sapiens was set as the study species to obtain and integrate information on the potential targets of DNJ and TFs. For Swiss Target Prediction, only targets with a probability greater than 0.1 were retained. For Pharm Mapper, targets with a normalized fit value greater than 0.6 were selected.

The GeneCards (https://www.genecards.org, accessed on 11 November 2021), DisGeNET (https://www.disgenet.org/, accessed on 11 November 2021), and OMIM (https://omim.org/, accessed on 11 November 2021) databases were searched for the keyword “diabetes mellitus type 2 (T2DM)” to find the genes related to T2DM, and to construct a dataset of disease target information for T2DM. Standardization was performed through the Uniprot database (https://www.uniprot.org/, accessed on 15 November 2021).

### 2.12. Constructing Protein–Protein Interaction (PPI) Network

The “DNJ, TFs-T2DM-targets” were then subjected to analysis using the STRING database (https://string-db.org/, accessed on 20 November 2021) to construct a protein–protein interaction (PPI) network [20], with high confidence (0.7). The resulting PPI network was subsequently visualized with the aid of Cytoscape 3.8.0 software.

### 2.13. Gene Ontology (GO) and Kyoto Encyclopedia of Genes and Genomes (KEGG) Pathway Enrichment Analysis

The obtained DNJ, TFs-T2DM core targets were imported into the DAVID database (https://davidbioinformatics.nih.gov/, accessed on 20 November 2021), limiting the study species to Homo sapiens, and selecting Biological Processes (BP), Molecular Function (MF), Cellular Components (CC), and KEGG Pathway for enrichment and pathway analysis. The test level was set at *p* < 0.01 and finally visualized using R Studio 4.2.2.

### 2.14. Molecular-Docking Analysis

The structures of key tarobtain proteins in the PPI network were downloaded from the RCSB PDB database (http://www.rcsb.org/, accessed on 22 November 2021) and imported into Autodock 4.2.6., which were collected from the PubChem database (https://pubchem.ncbi.nlm.nih.gov/, accessed on 22 November 2021) to get the MDL Molfile (*.mol) format of DNJ and TFs, and then imported into ChemBio3D Ultra 14.0 software to convert the two-dimensional structure into a three-dimensional structure. It was imported into the docking software Autodock 4.2.6, and its energy was minimized and converted to *pdbqt format using the function in PyRx 0.8 software. The ligand and receptor constructed above were imported into Autodock software, and small molecule-protein docking was performed using the Vina module.

Docking scores less than −7.0 indicated that the active ingredient had a strong binding activity to the target, and scores less than −5.0 indicated that the two had a good binding activity.

### 2.15. AlphaLisa-Based Cellular Thermal Shift Assay (CETSA)

The HepG2 cells were divided into 1% DMSO group and DNJ-TFs treatment group. For AlphaLisa-based CETSA, cells were lysed in CETSA Cell Lysis Buffer 2 (PerkinElmer, Waltham, MA, USA). Reactions were completed in 384-well plates; each reaction was conducted with 10 μL final volume: 5 μL of lysate, and 5 μL of 1:800 rabbit anti-MMP9/PTGS2 (Proteintech Group, Shanghai, China) +1:100 anti-rabbit IgG MMP9/PTGS2 acceptor beads (PerkinElmer, USA) + AlphaLisa Immunoassay buffer (PerkinElmer, USA). AlphaLISA assays were performed using Envision Alpha Reader (Thermo Fisher Scientific, Waltham, MA, USA).

### 2.16. Immunofluorescence Staining

For the immunofluorescence study, cells were seeded in culture-grade glass coverslips. After treatment, the cells were fixed with 4% PFA for 10 min, permeabilized with 0.1% Triton X-100 (Beyotime, Shanghai, China), and then blocked with 5% BSA in TBST for 30 min at room temperature. The cells were then incubated with the MMP9 (1:100)/PTGS2 (1:100) primary antibodies (Proteintech Group, Shanghai, China) overnight at 4 °C. After PBS washes, the cells were incubated with secondary antibodies for 1 h at room temperature. Following 3 washes with PBS, DAPI diluent (Beyotime, Shanghai, China) was used for nuclear counterstaining. Fluorescent images were captured on a confocal laser-scanning microscope (Leica TCS SP8)

### 2.17. Quantitative Real-Time PCR

Total RNA was isolated from cells with TRIzol reagent (Invitrogen, Carlsbad, CA, USA) according to the manufacturer’s instructions. Total RNA was reverse transcribed to cDNA using a High-Capacity cDNA Reverse Transcription Kit (Applied Biosystems, Foster City, CA, USA). Real-time PCR was performed with the SYBR Green PCRMaster Mix using a StepOnePlus Real-Time PCR System (Servicebio, Wuhan, China). The expression of actin was used as an internal control. All primers were synthesized by Sangon Biotech (Shanghai, China), and the sequences are shown in Supplemental Table S1.

### 2.18. Western Blot

A Western blot was performed as described in [21]. The primary antibodies used were as follows: GAPDH (1:10,000), MMP9 (1:1000), RANKL (1:1000), TNF-α (1:1000), AKT (1:2000), *p*-AKT (Ser473) (1:2000), GLUT2 (1:1000), PTGS2 (1:600), cAMP (1:1000), GSK 3α (1:3000), GSK 3β (1:2000), *p*-GSK 3α (Ser21) (1:2000), and *p*-GSK 3β (Ser9) (1:2000). The secondary antibody was goat anti-rabbit IgG (1:8000). All antibodies were purchased from Prteintech Group (Shanghai, China).

### 2.19. Statistical Analysis

Values are presented as mean SD. All data shown were representative of at least three individual experiments. Statistical analyses were performed with SPSS 25 software (SPSS, Chicago, IL, USA). Significance was determined by using a two-tailed Student’s s-test or one-way ANOVA as appropriate. *p*-values of <0.05 were considered significant.

## 3. Results

### 3.1. Synergistic Hypoglycemic Ability of DNJ and TFs

**Figure 1:**
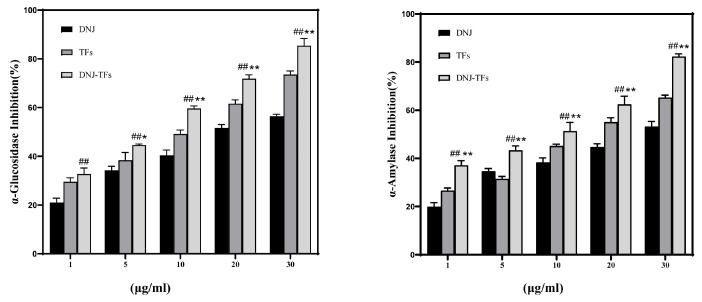
**DNJ and TFs synergistically inhibit α-glucosidase and α-amylase.** Inhibitory effects of different concentrations of DNJ, TFs, and their equal proportion mixture on α-glucosidase and α-amylase. DNJ—1-Deoxynojirimycin; TFs—Theaflavin. * *p* < 0.05 and ** *p* < 0.01 vs. TFs; ## *p* < 0.01 vs. DNJ.

As shown in Figure 1, the inhibition rates of α-glucosidase and α-amylase after DNJ combined with TFs administration were higher than those of any single administration group, and the differences were maintained at concentrations of 5 μg/mL and above. The synergistic hypoglycemic ability of DNJ-TFs was evaluated based on the Chou–Talalay model. Table 1 shows the half-maximal inhibitory concentration (IC50) values for α-glucosidase inhibition rate and α-salivary amylase inhibition rate of DNJ, which were 27.01 μg/mL and 22.69 μg/mL, respectively, while those for TFs were 16.13 μg/mL and 12.46 μg/mL, respectively. For the combination of DNJ-TFs, the IC50 values for α-glucosidase inhibition rate and α-salivary amylase inhibition rate were 10.93 μg/mL and 11.01 μg/mL, respectively. Upon inputting these values into the Chou–Talalay model calculation software “CompuSyn” (ComboSyn Inc., Paramus, NJ, USA), the combined effect indices (CIs) were 0.85 and 0.76, both below 1. This indicates that DNJ and TFs exhibit synergistic inhibitory effects against α-glucosidase and α-salivary amylase.

Meanwhile, Supplementary Figure S1 shows that when the DNJ:TF concentration ratio was 2:1, the α-glucosidase inhibition rate was optimal; this ratio will also be used in the subsequent cell and animal experiments. Subsequently, we analyzed the data from the α-glucosidase inhibition experiment using two-way ANOVA, and the results further confirmed the synergistic hypoglycemic effect of DNJ and TFs (Supplementary Table S2).

### 3.2. DNJ and TFs Improve PA-Induced IR in HepG2 Cells

According to Supplementary Figure S2 and the optimal ratio, a mixture of 10 μg/mL DNJ solution and 5 μg/mL TFs solution was selected as the low-concentration treatment group (Group A), a mixture of 20 μg/mL DNJ solution and 10 μg/mL TFs solution was selected as the high-concentration treatment group (Group B), and 300 μM PA was selected for subsequent experiments.

**Figure 2:**
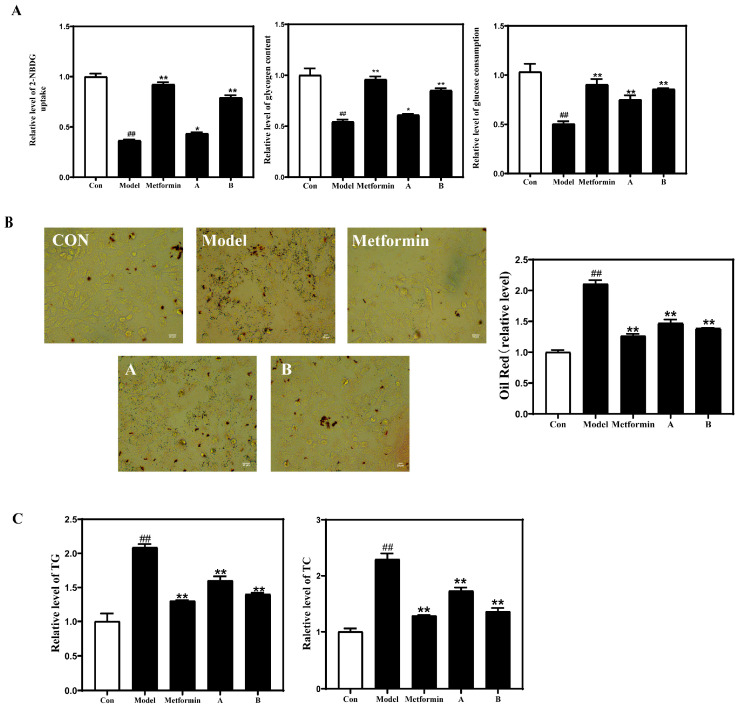
**DNJ and TFs improve PA-induced IR in HepG2 cells.** (**A**) Changes in glucose uptake, glycogen content, and glucose consumption in differently treated HepG2 cells; Lipid accumulation (**B**), total cholesterol (TC), and serum level of triglyceride (TG) content (**C**) in HepG2 cells after different treatments. DNJ—1-Deoxynojirimycin; TFs—Theaflavin; IR—insulin resistance. Con—control group; Model—300 μM PA for 24 h; Metformin—300 μM PA + 10 μg/mL metformin for 24 h; A—300 μM PA + 10 μg/mL DNJ + 5 μg/mL TFs for 24 h; B—300 μM PA + 20 μg/mL DNJ + 10 μg/mL TFs for 24 h. * *p* < 0.05 and ** *p* < 0.01 vs. Model; ## *p* < 0.01 vs. Con.

As expected, IR occurred in HepG2 cells treated with PA, which was reflected in the decrease in glucose uptake, glucose consumption, and intracellular glycogen content in the Model group (Figure 2A). In addition, lipid accumulation and TC and TG contents in the cells were significantly increased in the Model group compared with the Con group (Figure 2B,C). However, all PA-induced changes were inhibited after IR cells were treated with the positive control drug metformin and a mixture of different concentrations of DNJ-TFs (Figure 2).

### 3.3. DNJ-TFs Improve IR in HFD-Induced T2DM Mice

**Figure 3:**
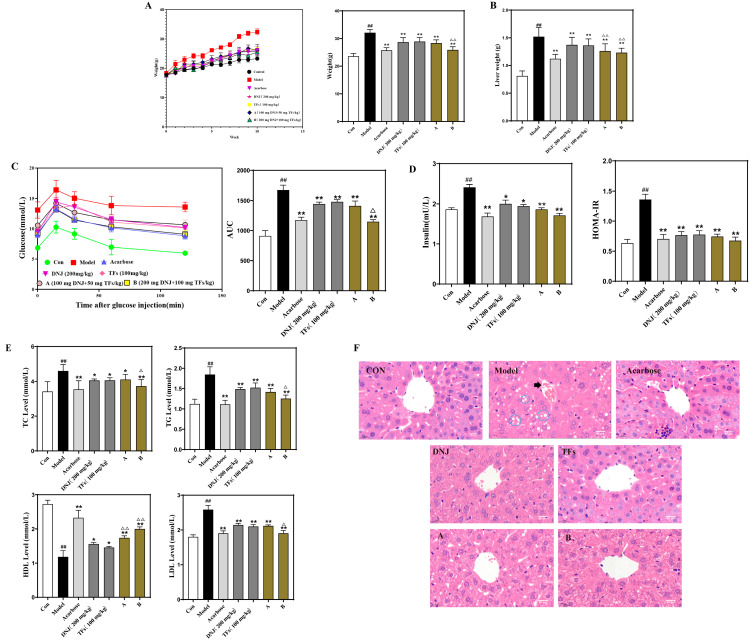
**DNJ-TFs improve IR in high-fat-induced T2DM mice.** Male mice were fed a normal diet or a high-fat diet and given or not given 10 mg/mL acarbose and different concentrations of DNJ and TFs dissolved in drinking water. (**A**) Body weight changes during the experiment and mean body weights at week 10; (**B**) liver weights; (**C**) the changes in blood glucose during IPGTT and the AUC of glucose between 0 and 120 min of IPGTT; (**D**) the levels of serum insulin, HOMA-IR values; (**E**) total cholesterol (TC), serum level of triglyceride (TG), low-density lipoprotein cholesterol (LDL) and high-density lipoprotein cholesterol (HDL); (**F**) HE staining of liver tissue (scale bars, 50 μm) of mice of different groups. DNJ—1-Deoxynojirimycin; TFs—Theaflavin; IR—insulin resistance. A—high-fat + 100 mg/kg DNJ + 50 mg/kg TFs; B—high-fat + 200 mg/kg DNJ + 100 mg/kg TF. * *p* < 0.05 and ** *p* < 0.01 vs. Acarbose; ## *p* < 0.01 vs. Con; ^△^ *p* < 0.05 and ^△△^ *p* < 0.01 vs. DNJ (20 mg/mL) and TFs (10 mg/mL).

Compared with the Con group, HFD feeding significantly increased the body weight and liver weight of the mice (average body weight was 33.6 g, and average liver weight was 1.53 g), while the positive drugs Acarbose, DNJ, TFs, and different concentrations of DNJ-TFs all inhibited the weight changes caused by HFD feeding. The inhibitory effect of high concentrations of DNJ-TFs mixed feeding was better than that of DNJ and TFs alone (Figure 3A,B).

At the end of the experiment, all mice were subjected to IPGTT. The AUC values of glucose at 0 and 120 min were calculated. The results showed that the AUC value of the Model group was significantly higher than that of the Con group, while the AUC values of the Acarbose group, DNJ group, TFs group, and DNJ-TFs mixed group A and group B were significantly lower than the Model group. The AUC of the DNJ-TFs hybrid group B decreased by approximately 30% compared to the model group. In addition, HOMA-IR values were significantly higher in the Model group compared to the Con group, but acarbose, DNJ, TFs, and DNJ-TFs combination therapy all significantly inhibited half of the changes in HOMA-IR value in the model group (Figure 3C,D). In addition, two-way ANOVA further demonstrated the synergistic hypoglycemic effect of DNJ and TFs (Supplementary Table S3). Figure 3E shows the changes in blood lipids in mice, and the results showed that both acarbose and the combined treatment of high concentrations of DNJ-TFs inhibited the changes in blood lipids induced by HFD feeding, although the effects induced by DNJ and TFs alone were not significant. In addition, histopathological analysis of the liver showed that the liver cytoarchitecture of mice in the Model group was deformed, and hepatic sinusoidal sludge and extensive steatosis (ellipse marking) with inflammatory cell infiltration (arrow marking) were seen. Hepatocyte lipoatrophy and inflammatory cell infiltration were reduced in mice in all dosing groups (Figure 3F and Supplementary Table S4).

### 3.4. Exploring Candidate Targets of DNJ-TFs in Improving T2DM Through Network Pharmacology

**Figure 4:**
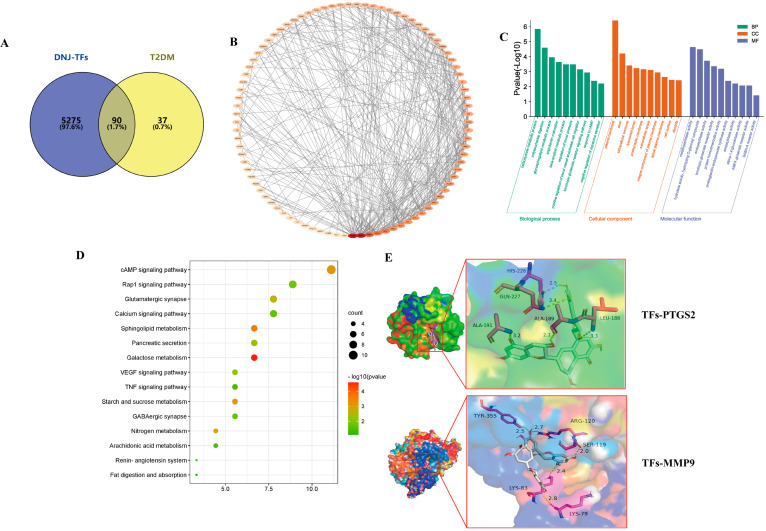
**Network pharmacology explores candidate targets of DNJ-TFs for improving type 2 diabetes (T2DM).** (**A**) Intersection of potential targets of DNJ-TFs and potential targets of T2DM; (**B**) DNJ-TFs anti-T2DM protein–protein interaction (PPI) network; (**C**) gene ontology (GO) analysis bar chart of core genes; (**D**) Kyoto Encyclopedia of Genes and Genomes (KEGG) enrichment bubble map of core genes; (**E**) molecular docking results of TFs-PTGS2 and TFs-MMP9. TFs—Theaflavin.

After cross-checking/merging and deduplication of databases, we finally obtained 127 potential targets of DNJ-TFS and 5365 T2DM-related target information. The two genomes were intersected through a Venn diagram, and 90 targets for DNJ-TFs combined anti-T2DM were obtained (Figure 4A). The genes were imported into the String database, and the PPI network was constructed as shown in Figure 4B. The important targets in the PPI network diagram include PTGS2, VEGFA, MMP9, DRD2, ACE, and GLB1. Network Analyzer was used to perform topological parameter analysis, obtain degree centrality, and screen targets with a value higher than the average value of 8.06 as key targets, and a total of 36 key targets were obtained (Supplementary Table S5). Subsequently, we performed functional enrichment analysis and KEGG signaling pathway enrichment on the 36 core target genes. The GO analysis results obtained 23 cellular components, 30 molecular functions, and 82 biological processes, and the top 10 T2DM-related indicators were visualized according to the *p* value (Figure 4C). The top two biological processes included the carbohydrate metabolic process and polysaccharide digestion. The top two cellular components were the plasma membrane and the axon. The molecular function mainly focused on the metallopeptidase activity and hydrolase activity, hydrolyzing O-glycosyl compounds. KEGG analysis showed that DNJ-TFs anti-T2DM targets were mainly enriched in the cAMP signaling pathway, Rap1 signaling pathway, Calcium signaling pathway, VEGF signaling pathway, TNF signaling pathway, etc. (Figure 4D).

Molecular docking results showed TFs-PTGS2 and TFs-MMP9 had the best binding activity, and the binding energies of the two groups reached −10.00 kcal/mol and −8.90 kcal/mol, and both generated hydrogen bonds, as shown in Figure 4E. In addition, the binding energies of DNJ with PTGS2 and MMP9 were −5.7 kcal/mol and −5.3 kcal/mol, respectively, which were both less than −5.0, indicating that PTGS2 and MMP9 might be the key target genes for DNJ-TFs to synergistically lower blood sugar levels.

### 3.5. PTGS2 and MMP9 Are Target Genes of DNJ-TFs

**Figure 5:**
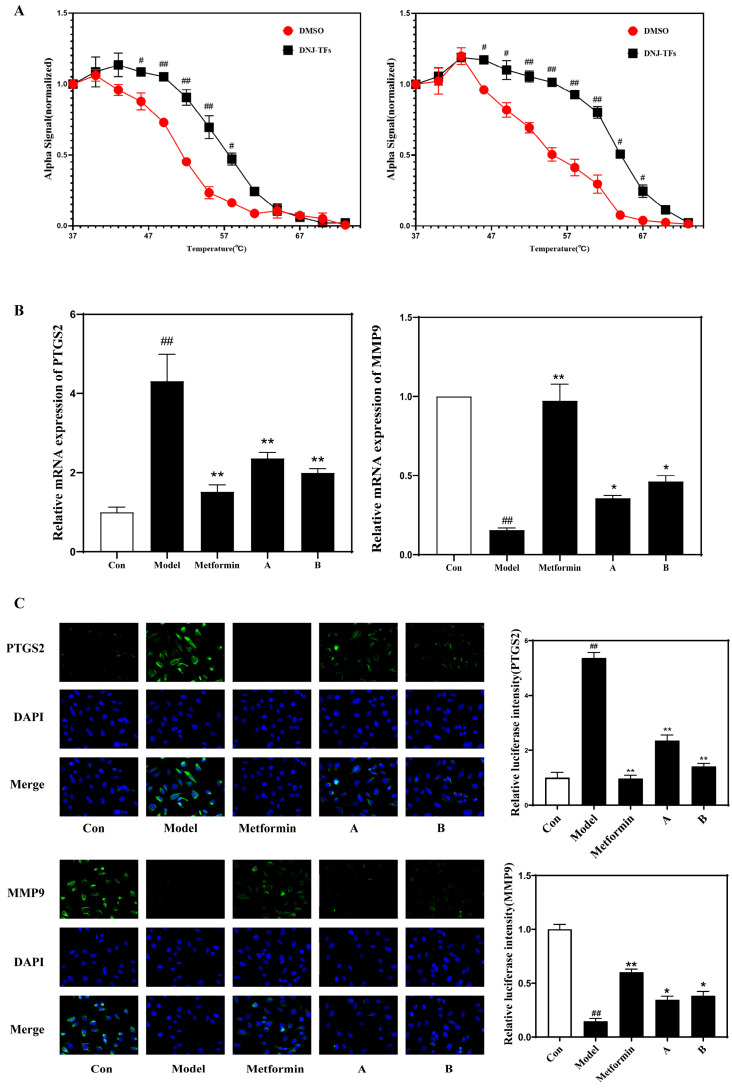
**PTGS2 and MMP9 are target genes of DNJ-TFs.** (**A**) Determination of thermal displacement of PTGS2 and MMP9 in HepG2 cells at different temperatures; expression of PTGS2 and MMP9 mRNA (**B**) and immunofluorescence intensity (**C**) in HepG2 cells of each group. Con—control group; Model—300 μM PA for 24 h; Metformin—300 μM PA + 10 μg/mL metformin for 24 h; A—300 μM PA + 10 μg/mL DNJ + 5 μg/mL TFs for 24 h; B—300 μM PA + 20 μg/mL DNJ + 10 μg/mL TFs for 24 h. * *p* < 0.05 and ** *p* < 0.01 vs. Model; # *p* < 0.05 and ## *p* < 0.01 vs. Con.

CETSA technology was used to detect the affinity between DNJ-TFs and MMP9 and PTGS2. Figure 5A shows that, in the range of 37~65 °C, the MMP9 and PTGS2 shift curves of the DNJ-TFs group were significantly shifted to the right compared with the 1% DMSO group, and there was a significant difference starting at 46 °C, suggesting that DNJ-TFs and MMP9 and PTGS2 combine to improve their thermal stability. After the corresponding treatment was given to HepG2 cells, the real-time PCR and immunofluorescence results showed that PA treatment increased the mRNA and fluorescence intensity of PTGS2 in the cells and reduced the mRNA and fluorescence intensity of MMP9. Treatment with positive control drugs and high concentrations of DNJ-TFs can suppress more than half of this phenomenon. Meanwhile, overexpression of PTGS2 and inhibition of MMP9 suppressed the effect of DNJ-TFs in alleviating PA-induced hepatocyte damage, suggesting that PTGS2 and MMP9 were target genes for DNJs and TFs acting together (Figure 5B,C and Supplementary Figure S3).

### 3.6. DNJ-TFs Improve HFD-Induced Inflammation and IR by Targeting PTGS2 or MMP9 via the TNFα/AKT/GSK3/GLUT2 Signaling Pathway

**Figure 6:**
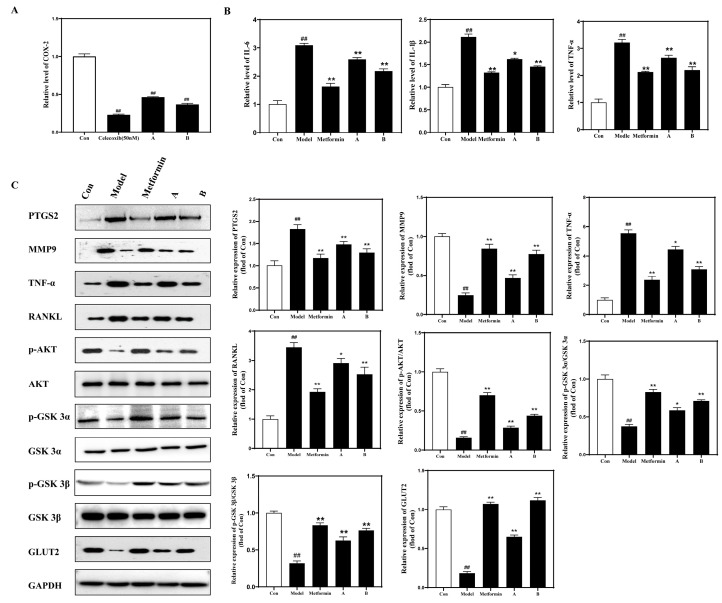
**DNJ-TF targets PTGS2 or MMP9 in HepG2 cells and improves PA-induced inflammation and insulin resistance via the TNFα/AKT/GSK3/GLUT2 signaling pathway.** (**A**) Inhibition rate of COX-2 by DNJ-TFs; (**B**) expression of inflammatory factors IL-6, IL-1β, and TNF-α in HepG2 cells in each group; (**C**) protein expressions of PTGS2, MMP9, TNF-α, RANKL, *p*-AKT/AKT, *p*-GSKα/GSKα, *p*-GSKβ/GSKβ, and GLUT2 in HepG2 cells of each group. Model—300 μM PA for 24 h; Metformin—300 μM PA + 10 μg/mL metformin for 24 h; A—300 μM PA + 10 μg/mL DNJ + 5 μg/mL TFs for 24 h; B—300 μM PA + 20 μg/mL DNJ + 10 μg/mL TFs for 24 h. * *p* < 0.05 and ** *p* < 0.01 vs. Model; ## *p* < 0.01 vs. Celecoxib/Con.

**Figure 7:**
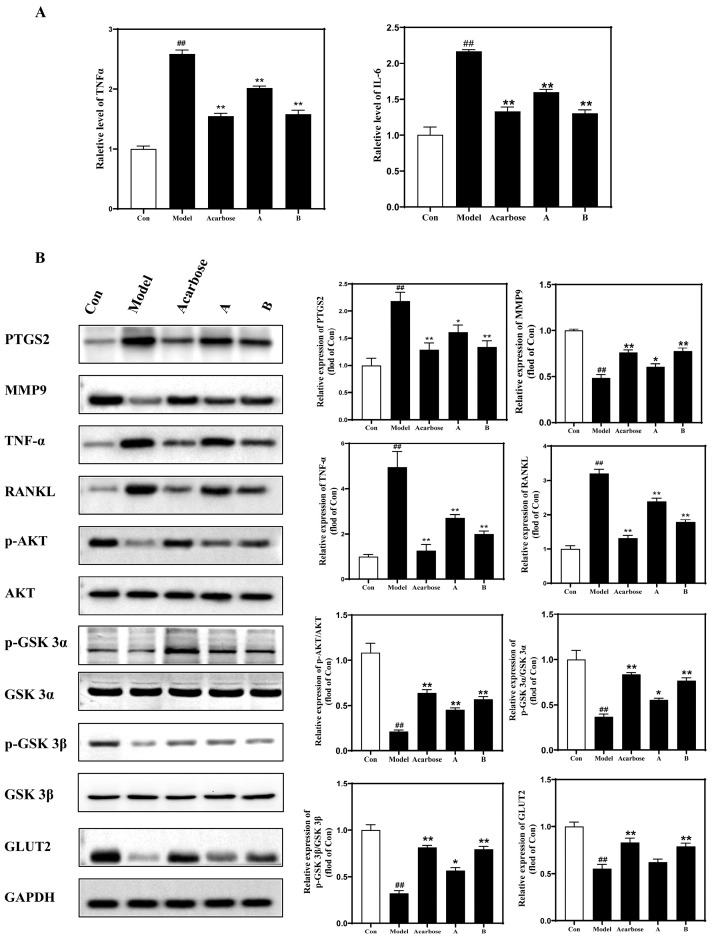
**DNJ-TF targets PTGS2 or MMP9 in mouse liver and improves high-fat feeding-induced inflammation and insulin resistance via the TNFα/AKT/GSK3/GLUT2 signaling pathway.** (**A**) Expression of inflammatory factors IL-6 and TNF-α in serum in each group; (**B**) protein expressions of PTGS2, MMP9, TNF-α, RANKL, *p*-AKT/AKT, *p*-GSKα/GSKα, *p*-GSKβ/GSKβ, and GLUT2 in liver tissue of each group. A—high-fat + 100 mg/kg DNJ + 50 mg/kg TFs; B—high-fat + 200 mg/kg DNJ + 100 mg/kg TFs. * *p* < 0.05 and ** *p* < 0.01 vs. Model; ## *p* < 0.01 vs. Con.

The results of the COX inhibition experiment showed that both concentrations of DNJ-TFs, A and B, had the ability to antagonize COX-2, and the effect of the higher dose of DNJ-TFs was similar to that of the positive control, celecoxib, with the inhibition rate reaching more than half (Figure 6A). Meanwhile, as the duration of PA intervention increased, the number of inflammatory factors in hepatocytes also gradually increased (Supplementary Figure S4). Subsequently, by detecting changes in inflammatory markers and oxidative stress factors in cells, mouse serum, and liver tissue, it was found that the positive control drug and different concentrations of DNJ-TFs could improve the changes in inflammatory markers and oxidative stress factors induced by a high-fat diet, with higher concentrations of DNJ-TFs showing more significant effects. (Figure 6B and Figure 7A, (Supplementary Figures S5 and S6). Finally, we detected the target genes of DNJ-TFs and the key proteins of inflammation and insulin signaling pathways. The results showed that DNJ-TFs could inhibit the increase in PTGS2, TNFα, RANKL, and the decrease in MMP9, *p*-AKT/AKT, *p*-GSK-3α/GSK-3α, *p*-GSK-3β/GSK-3β, and GLUT2 induced by high fat both in vivo and in vitro experiments, and the effect of a high concentration of DNJ-TFs was close to that of positive drugs (Figure 6C and Figure 7B).

## 4. Discussion

Traditional Chinese medicine theory suggests that mulberry leaves and black tea can be used together to improve IR while clearing away heat and moistening dryness, regulating cold and heat, and avoiding excessive cold that hurts the stomach. Based on this, this study will explore the synergistic hypoglycemic effect and mechanism of DNJ and TFs for the first time. Recently, the use of bioactive α-glucosidase inhibitors and α-amylase inhibitors for the treatment of diabetes has been shown to be an effective remedy for controlling postprandial hyperglycemia and its deleterious physiological complications. The carbohydrate hydrolase α-glucosidase is often competitively inhibited by α-glucosidase inhibitors, resulting in delayed intestinal glucose absorption and ultimately controlling postprandial hyperglycemia [22]. Similarly, blood glucose levels can be modulated through the inhibition of α-amylase, which is involved in the digestion of starch and disaccharides [23]. In this study, we demonstrated that DNJs and TFs have synergistic anti-glycemic effects using the α-glucosidase inhibition assay as well as the α-amylase inhibition assay. More importantly, the dual inhibition of α-glucosidase and α-amylase not only delayed cellular glucose uptake but also helped improve intracellular insulin signaling. By reducing postprandial blood glucose spikes, it alleviated metabolic stress in insulin-sensitive tissues, thereby weakening the sustained activation of negative regulators such as JNK. This relief promoted phosphorylation of the tyrosine residues of insulin receptor substrate-2 (IRS-2) and enhanced the activity of the downstream PI3K/Akt pathway, thereby improving GLUT2 translocation and glucose uptake in tissues [24,25]. The Chou–Talalay method is considered to be the most influential method for quantifying synergism [26]. Using this method, we further confirmed the presence of a synergistic effect between DNJ and TFs.

After exploring the optimal treatment concentrations and ratios of the two, PA was used to construct a hepatocellular IR model in vitro as well as an in vivo mouse model of T2DM induced by an HFD, where positive controls and mixtures of DNJ-TFs were given to assess the synergistic effects of DNJ-TFs on diabetes. Both in vivo and in vitro results showed that DNJ-TFs ameliorated high-fat-induced impaired glucose metabolism-related indices and increased lipids in cells and mice serum.

DNJ and TFs exert a synergistic hypoglycemic effect. Besides inhibiting α-glucosidase and α-amylase, TFs can also inhibit sodium–glucose cotransporters or regulate intestinal glucose transporters such as GLUT2, thereby reducing glucose uptake by the intestinal epithelium. DNJ, by delaying carbohydrate digestion, provides a dual blockade, resulting in a slower rise in postprandial blood glucose. Both also possess anti-inflammatory and antioxidant effects [27,28]. Therefore, in order to explore the detailed mechanism of DNJ-TFs synergizing hypoglycemic effect, through network pharmacology methods and experimental verification, it was finally determined that DNJ and TFs acted together on the target genes PTGS2 and MMP9.

PTGS2 is known as prostaglandin endoperoxidase synthase 2, also known as cyclooxygenase 2 (COX2). It is tightly regulated and usually expressed at very low levels under physiological conditions, and its expression and activation are directly induced by pro-inflammatory cytokines and growth factors that activate intracellular inflammation-related pathways [29,30]. It is mainly involved in inflammation-related processes and has been described as a potential marker of pathology showing high levels of inflammation [31]. The pro-inflammatory state driven by PTGS2 and its metabolite prostaglandin E2 (PGE2) can maintain the activation of stress kinases such as JNK. JNK can phosphorylate inhibitory serine residues of IRS, which is a key linker protein in the insulin signaling cascade. This further impairs its function and promotes its degradation, thereby reducing AKT phosphorylation levels and reducing insulin sensitivity [32]. In this study, the high-affinity binding of DNJ-TFs to PTGS2 likely blocked its driving process, thereby inhibiting PGE production and alleviating inflammation-driven IR. Studies have shown that TFs can reduce the expression of PTGS2 in the brain tissue of rats with cerebral ischemia and in the serum of patients with colon cancer [33,34], and DNJ can reduce the expression of PTGS2 in cancer cells [35], which is similar to our results. In addition, Johanna et al. found that T2DM in older men was associated with PTGS2-mediated inflammation [36]; Yasmine et al. found that PTGS2-mediated inflammation may contribute to the development of T2DM in some individuals by examining the correlation between PTGS2 promoter variants and T2DM [37]. Meanwhile, Hsieh et al. found that a PTGS2 inhibitor could significantly reverse the time-dependent increase in plasma insulin, glucose, and HOMA-IR in high-fat-induced obese rats [38]. This suggests that DNJ-TFs are likely to inhibit high-fat-induced IR by targeting PTGS2. However, the implications of inhibiting this key enzyme likely extend far beyond the hepatic tissue. PTGS2, as the rate-limiting enzyme in PGE2 synthesis, is located upstream in multiple neuroinflammatory cascades [39], and under metabolic stress, the liver becomes a significant source of circulating inflammatory mediators. Hepatic PTGS2 activity-driven PGE2 and other cytokines disrupt the integrity of the blood–brain barrier and directly activate microglia—the brain’s innate immune cells [40]. This initiates a positive feedback loop of neuroinflammation characterized by the production of central cytokines such as TNF-α and IL-1β, which subsequently impair hypothalamic insulin signaling and further exacerbate the inflammatory response, disrupting central regulation of energy balance and glucose homeostasis [41].

Matrix metalloproteinases (MMPs) are a family of zinc enzymes responsible for the degradation and remodeling of the extracellular matrix proteins during normal developmental processes, such as organ morphogenesis and angiogenesis in pathological processes, such as inflammation and tumor invasion [42]. The binding we observed may be that DNJ-TFs interfere with the protease function of MMP9 by occupying the zinc ion-binding domain or other key regions, thereby delaying the progression of diabetes [43]. MMP9 is the largest MMP, primarily secreted by neutrophils and macrophages. Elevated MMP9 levels have been shown to directly cleave and degrade IRS-1. IRS-1 deficiency severely interferes with subsequent activation of the PI3K/Akt pathway, thereby impairing insulin metabolism [44]. Its role in inflammation is particularly complex and context-dependent, making it a double-edged sword [45]. On the one hand, there is evidence to support its anti-inflammatory functions. For example, Wan et al. found significant increases in inflammatory markers, such as RANKL, IL-1β, and TNF-α, in apical lesions of MMP-9 KO mice. MMP9 has also been shown to release soluble fragments of the integrin β2 subunit from the surface of monocytes/macrophages into the extracellular milieu, acting as a receptor antagonist and thus limiting local inflammation [46,47]. On the other hand, it can degrade tight junction proteins, disrupt the integrity of the blood–brain barrier, and activate potential pro-inflammatory cytokines, such as TNF-α and IL-1β, thereby amplifying the inflammatory cascade and leading to tissue damage [48]. Therefore, given this duality, whether DNJ-TFs exert their effects by simply activating MMP9 or by modulating the pathological environment to enable MMP9 to perform anti-inflammatory and reparative functions remains to be determined.

The key genes of DNJ-TFs against T2DM were identified by network pharmacology and enriched the KEGG signaling pathway, among which the tumor necrosis factor (TNF) signaling pathway attracted our attention. TNF is a cytokine that has pleiotropic effects on various cell types. It not only induces its own secretion, but it also stimulates the production of other inflammatory cytokines and chemokines, and is known to be involved in the pathogenesis of some inflammatory and autoimmune diseases [49]. Receptor activator of nuclear factor κB Ligand (RANKL) is a member of the TNF superfamily and is secreted by osteoblasts, bone marrow stromal cells, and lymphocytes [50]. Studies have shown that TNFα promotes the expression of RANKL and participates in the inflammatory process [42]. At the same time, our key gene, PTGS2, is also shown to promote the activation of the TNF signaling pathway, while MMP9 is just the opposite [42,51], which once again confirmed our hypothesis: DNJ-TFs affect the occurrence of inflammation by acting on PTGS2 and MMP9.

Accumulating evidence corroborates the crucial role of inflammation in T2DM pathologies. Increased levels of inflammatory markers are believed to be associated with the pathogenesis of T2DM [52]. At the same time, TNF-α is believed to further stimulate the AKT pathway [53,54]. The mechanism may involve TNFα activation of cellular stress kinases, such as JNK and IKKβ, which in turn phosphorylate inhibitory serine residues of IRS-2 [55,56]. This post-translational modification impairs the function of IRS-2, a key adaptor protein that recruits and activates PI3K upon insulin receptor activation. Impaired IRS-2 leads to incomplete PI3K activation, resulting in reduced AKT phosphorylation levels and its downstream metabolic effects on glucose homeostasis and lipid metabolism [57,58]. Akt is one of the main signaling pathways for insulin to regulate metabolic function and is involved in glycolipid metabolism in a variety of cells [17]. The changes in systemic insulin sensitivity observed in our model were mainly mediated by the AKT2 subtype, which is considered to be the main subtype for insulin delivery in peripheral tissues such as the liver and skeletal muscle. AKT1 plays a key role in promoting cell growth and regulating apoptosis, while AKT3 plays a more significant role in regulating neurodevelopment and brain function [59]. Glycogen synthase kinase-3 (GSK-3), as a downstream effector of the AKT pathway, may be crucial for inhibiting the inflammatory environment and restoring classical insulin-mediated glycogen synthesis. Meanwhile, GSK3α inhibition may be particularly beneficial in alleviating high-fat diet-induced hepatic steatosis by inhibiting de novo adipogenesis and further promoting glycogen storage [60,61]. Glucose transporters 2 (GLUT2), a member of this family of GLUTs, mediates passive transmembrane transport of glucose, during which it interacts with cells of multiple organs involved in glucose metabolism. The transport activity of GLUT2 controls the expression of genes involved in mechanisms that regulate metabolic pathways and maintain glucose homeostasis in cells [62]. GLUT2 is also a downstream effector of AKT, and it has been claimed that GSK-3 inhibitors reduce GLUT2 expression [63,64]. It is worth noting that the downregulation of hepatic GLUT2 expression observed in our study can be explained as a downstream result of weakened AKT signaling pathway and direct transcriptional inhibition of TNFα through the NF-κB pathway [65,66]. Ultimately, this leads to liver metabolic dysfunction. In our study, we found that DNJ-TFs reduced the expression of high-fat-induced inflammatory factors and inhibited high-fat-induced reduction in *p*-AKT, *p*-GSK3α, *p*-GSKSβ, and GLUT2. This suggests that DNJ-TFs can improve T2DM by ameliorating the high-fat-induced increase in inflammation, impaired insulin signaling pathway, decreased glycogen synthesis, and impaired glucose transport.

In addition to its role in hepatic insulin resistance, GSK3 is increasingly recognized as a pathological node in a variety of age-related diseases, particularly neurodegenerative diseases such as Alzheimer’s disease (AD) [67]. In the brain, GSK3β overactivity drives core features of AD pathology, including tau protein hyperphosphorylation, leading to neurofibrillary tangle formation and promoting β-amyloid production [68]. Therefore, our study elucidates a novel up-mechanism leading to GSK3β dysregulation, providing important clues to the pathogenesis of AD. A growing body of literature emphasizes the existence of a bidirectional “liver-brain axis,” in which peripheral metabolic inflammation can interfere with central insulin signaling, and vice versa [69,70]. We observed that DNJ-TFs in the liver inhibited systemic inflammatory mediator levels via PTGS2/MMP9. These circulating factors have been shown to disrupt the integrity of the blood–brain barrier and directly inhibit insulin receptor signaling in key brain regions, such as the hypothalamus [41]. This central insulin resistance impairs the anorexia and metabolic effects of insulin in the brain, creating a self-reinforcing vicious cycle leading to systemic metabolic dysfunction. Therefore, DNJ-TFs not only have therapeutic value in improving hepatic insulin resistance but may also help mitigate the potentially detrimental effects of insulin resistance on central nervous system function.

Meanwhile, this study has some limitations. Since web-based pharmacology research relies on public databases, although we chose a database that is one of the most widely used platforms for Chinese medicine research, with a good reputation, and a large amount of data, we may still have missed some unlisted studies. Although existing studies support that PTGS2 and MMP9 affect IR and even T2DM by affecting inflammatory responses, further in-depth exploration and verification of the detailed mechanism are needed, which will also become our main focus for future research. Finally, while our findings elucidate the key mechanisms by which DNJ and TFs contribute to hepatic insulin resistance, the potential neurological relevance remains unexplored. Future studies should incorporate analyses of brain insulin signaling pathways or detection of neuroinflammatory markers to provide a more comprehensive understanding of the observed systemic effects.

## 5. Conclusions

The study showed that DNJ-TFs alleviate high-fat-induced inflammation and IR by targeting PTGS2 or MMP9 via the TNFα/AKT/GSK3/GLUT2 signaling pathway, and the synergistic anti-glycemic effect of the two drugs has a synergistic hypoglycemic effect, which provides new clues for the prevention and treatment of high-fat-induced T2DM and a new idea for the development of glucose-lowering products.

## Figures and Tables

**Table 1:** IC50 and CI values of DNJ, TFs, and DNJ-TFs.

α-Glucosidase Experiment		α-Amylase Experiment	
Group	IC_50_ (μg/mL)	Group	IC_50_ (μg/mL)
DNJ	27.01	DNJ	22.69
TFs	16.13	TFs	12.46
DNJ-TFs	10.93	DNJ-TFs	11.01
CI = 0.85		CI = 0.76	

CI—combined effect index; IC_50_—half-maximal inhibitory concentration.

## Data Availability

The data used and/or analyzed during the current study are available from the corresponding author upon reasonable request.

## Supplementary Materials

The following supporting information can be downloaded at: https://www.mdpi.com/article/10.3390/nu18010099/s1, Table S1: Primers used for qPCR analysis; Table S2: Two-way ANOVA of α-Glucosidase inhibition assay; Table S3: Two-way ANOVA of the AUC in the IPGTT; Table S4: NAS activity score; Table S5: Topological parameters of 36 key targets of DNJ-TFs against type 2 diabetes; Figure S1: α-Glucosidase inhibition rate of DNJ-TFs at different concentration ratios; Figure S2: Effects of DNJ, TFs and PA on HepG2 cell proliferation detected by CCK-8 assay; Figure S3: The role of DNJ-TFs in hepatocytes after overexpression of PTGS2 and inhibition of MMP9; Figure S4: Dynamic changes of inflammatory factors at different PA intervention times; Figure S5: Changes in inflammatory factors in liver tissue; Figure S6: Expression levels of oxidative stress factors in mice and HepG2 cells in each group.

## References

[1] Magliano D.J., Boyko E.J. (2021). IDF Diabetes Atlas.

[2] Ye C., Li Y., Shi J., He L., Shi X., Yang W., Lei W., Quan S., Lan X., Liu S. (2025). Network pharmacology analysis revealed the mechanism and active compounds of jiao tai wan in the treatment of type 2 diabetes mellitus via SRC/PI3K/AKT signaling. J. Ethnopharmacol..

[3] Zheng Y., Ley S.H., Hu F.B. (2018). Global aetiology and epidemiology of type 2 diabetes mellitus and its complications. Nat. Rev. Endocrinol..

[4] American Diabetes A. (2020). 11. Microvascular Complications and Foot Care: Standards of Medical Care in Diabetes-2020. Diabetes Care.

[5] Jedlowski P.M., Te C.H., Segal R.J., Fazel M.T. (2019). Cutaneous Adverse Effects of Diabetes Mellitus Medications and Medical Devices: A Review. Am. J. Clin. Dermatol..

[6] Zhang J., Zhu Y., Si J., Wu L. (2022). Metabolites of medicine food homology-derived endophytic fungi and their activities. Curr. Res. Food Sci..

[7] Ren X., Guo Q., Jiang H., Han X., He X., Liu H., Xiu Z., Dong Y. (2023). Combinational application of the natural products 1-deoxynojirimycin and morin ameliorates insulin resistance and lipid accumulation in prediabetic mice. Phytomedicine.

[8] Gothandam K., Ganesan V.S., Ayyasamy T., Ramalingam S. (2019). Antioxidant potential of theaflavin ameliorates the activities of key enzymes of glucose metabolism in high fat diet and streptozotocin—Induced diabetic rats. Redox Rep..

[9] Xu S., Chen Y., Gong Y. (2024). Improvement of Theaflavins on Glucose and Lipid Metabolism in Diabetes Mellitus. Foods.

[10] Wang K., Wu J., Chen S., Zhao H., He P., Tu Y., Li B. (2022). Transcriptome analysis provides insight into the anti-diabetic mechanism of theaflavins in high-fat diet and streptozotocin-induced mice. Food Funct..

[11] Fang Y., Wang J., Cao Y., Liu W., Duan L., Hu J., Peng J. (2024). The Antiobesity Effects and Potential Mechanisms of Theaflavins. J. Med. Food.

[12] ElSayed N.A., Aleppo G., Aroda V.R., Bannuru R.R., Brown F.M., Bruemmer D., Collins B.S., Hilliard M.E., Isaacs D., Johnson E.L. (2023). 2. Classification and Diagnosis of Diabetes: Standards of Care in Diabetes-2023. Diabetes Care.

[13] Ning K., Zhao X., Poetsch A., Chen W.H., Yang J. (2017). Computational Molecular Networks and Network Pharmacology. Biomed. Res. Int..

[14] Franco R.R., Mota Alves V.H., Ribeiro Zabisky L.F., Justino A.B., Martins M.M., Saraiva A.L., Goulart L.R., Espindola F.S. (2020). Antidiabetic potential of Bauhinia forficata Link leaves: A non-cytotoxic source of lipase and glycoside hydrolases inhibitors and molecules with antioxidant and antiglycation properties. Biomed. Pharmacother..

[15] Podsedek A., Majewska I., Redzynia M., Sosnowska D., Koziolkiewicz M. (2014). In vitro inhibitory effect on digestive enzymes and antioxidant potential of commonly consumed fruits. J. Agric. Food Chem..

[16] Dewangan J., Tandon D., Srivastava S., Verma A.K., Yapuri A., Rath S.K. (2017). Novel combination of salinomycin and resveratrol synergistically enhances the anti-proliferative and pro-apoptotic effects on human breast cancer cells. Apoptosis.

[17] Wang Y., Zhao X., Zhang L., Yang C., Zhang K., Gu Z., Ding H., Li S., Qin J., Chu X. (2023). MicroRNA-34a Mediates High-Fat-Induced Hepatic Insulin Resistance by Targeting ENO3. Nutrients.

[18] Banerjee J., Roy S., Dhas Y., Mishra N. (2020). Senescence-associated miR-34a and miR-126 in middle-aged Indians with type 2 diabetes. Clin. Exp. Med..

[19] Liu H., Zhu R., Liu C., Ma R., Wang L., Chen B., Li L., Niu J., Zhao D., Mo F. (2017). Evaluation of Decalcification Techniques for Rat Femurs Using HE and Immunohistochemical Staining. Biomed. Res. Int..

[20] Szklarczyk D., Gable A.L., Nastou K.C., Lyon D., Kirsch R., Pyysalo S., Doncheva N.T., Legeay M., Fang T., Bork P. (2021). The STRING database in 2021: Customizable protein-protein networks, and functional characterization of user-uploaded gene/measurement sets. Nucleic Acids Res..

[21] Lu N., Li Y., Qin H., Zhang Y.L., Sun C.H. (2008). Gossypin up-regulates LDL receptor through activation of ERK pathway: A signaling mechanism for the hypocholesterolemic effect. J. Agric. Food Chem..

[22] Hossain U., Das A.K., Ghosh S., Sil P.C. (2020). An overview on the role of bioactive alpha-glucosidase inhibitors in ameliorating diabetic complications. Food Chem. Toxicol..

[23] Riyaphan J., Pham D.C., Leong M.K., Weng C.F. (2021). In Silico Approaches to Identify Polyphenol Compounds as alpha-Glucosidase and alpha-Amylase Inhibitors against Type-II Diabetes. Biomolecules.

[24] Ozcan U., Cao Q., Yilmaz E., Lee A.H., Iwakoshi N.N., Ozdelen E., Tuncman G., Gorgun C., Glimcher L.H., Hotamisligil G.S. (2004). Endoplasmic reticulum stress links obesity, insulin action, and type 2 diabetes. Science.

[25] Sears B., Perry M. (2015). The role of fatty acids in insulin resistance. Lipids Health Dis..

[26] Roell K.R., Reif D.M., Motsinger-Reif A.A. (2017). An Introduction to Terminology and Methodology of Chemical Synergy-Perspectives from Across Disciplines. Front. Pharmacol..

[27] Kwon O., Eck P., Chen S., Corpe C.P., Lee J.H., Kruhlak M., Levine M. (2007). Inhibition of the intestinal glucose transporter GLUT2 by flavonoids. FASEB J..

[28] Zang M., Xu S., Maitland-Toolan K.A., Zuccollo A., Hou X., Jiang B., Wierzbicki M., Verbeuren T.J., Cohen R.A. (2006). Polyphenols stimulate AMP-activated protein kinase, lower lipids, and inhibit accelerated atherosclerosis in diabetic LDL receptor-deficient mice. Diabetes.

[29] Dubois R.N., Abramson S.B., Crofford L., Gupta R.A., Simon L.S., Van De Putte L.B., Lipsky P.E. (1998). Cyclooxygenase in biology and disease. FASEB J..

[30] Klein T., Shephard P., Kleinert H., Komhoff M. (2007). Regulation of cyclooxygenase-2 expression by cyclic AMP. Biochim. Biophys. Acta.

[31] Martin-Vazquez E., Cobo-Vuilleumier N., Lopez-Noriega L., Lorenzo P.I., Gauthier B.R. (2023). The PTGS2/COX2-PGE(2) signaling cascade in inflammation: Pro or anti? A case study with type 1 diabetes mellitus. Int. J. Biol. Sci..

[32] Aguirre V., Uchida T., Yenush L., Davis R., White M.F. (2000). The c-Jun NH(2)-terminal kinase promotes insulin resistance during association with insulin receptor substrate-1 and phosphorylation of Ser(307). J. Biol. Chem..

[33] Cai F., Li C.R., Wu J.L., Chen J.G., Liu C., Min Q., Yu W., Ouyang C.H., Chen J.H. (2006). Theaflavin ameliorates cerebral ischemia-reperfusion injury in rats through its anti-inflammatory effect and modulation of STAT-1. Mediat. Inflamm..

[34] Gosslau A., En Jao D.L., Huang M.T., Ho C.T., Evans D., Rawson N.E., Chen K.Y. (2011). Effects of the black tea polyphenol theaflavin-2 on apoptotic and inflammatory pathways in vitro and in vivo. Mol. Nutr. Food Res..

[35] Zhang R., Zhang Y., Xin X., Huang G., Zhang N., Zeng Q., Tang L., Attaribo T., Lee K.S., Jin B.R. (2021). Dual-Targeting Antiproliferation Hybrids Derived from 1-Deoxynojirimycin and Kaempferol Induce MCF-7 Cell Apoptosis through the Mitochondria-Mediated Pathway. J. Nat. Prod..

[36] Helmersson J., Vessby B., Larsson A., Basu S. (2004). Association of type 2 diabetes with cyclooxygenase-mediated inflammation and oxidative stress in an elderly population. Circulation.

[37] Konheim Y.L., Wolford J.K. (2003). Association of a promoter variant in the inducible cyclooxygenase-2 gene (PTGS2) with type 2 diabetes mellitus in Pima Indians. Hum. Genet..

[38] Hsieh P.S., Jin J.S., Chiang C.F., Chan P.C., Chen C.H., Shih K.C. (2009). It has been re-uploaded. Obesity.

[39] Andreasson K. (2010). Emerging roles of PGE2 receptors in models of neurological disease. Prostaglandins Other Lipid Mediat..

[40] Erickson M.A., Banks W.A. (2018). Neuroimmune Axes of the Blood-Brain Barriers and Blood-Brain Interfaces: Bases for Physiological Regulation, Disease States, and Pharmacological Interventions. Pharmacol. Rev..

[41] Thaler J.P., Yi C.X., Schur E.A., Guyenet S.J., Hwang B.H., Dietrich M.O., Zhao X., Sarruf D.A., Izgur V., Maravilla K.R. (2012). Obesity is associated with hypothalamic injury in rodents and humans. J. Clin. Investig..

[42] Zhang H., Liu L., Jiang C., Pan K., Deng J., Wan C. (2020). MMP9 protects against LPS-induced inflammation in osteoblasts. Innate Immun..

[43] Vandooren J., Van den Steen P.E., Opdenakker G. (2013). Biochemistry and molecular biology of gelatinase B or matrix metalloproteinase-9 (MMP-9): The next decade. Crit. Rev. Biochem. Mol. Biol..

[44] Banerjee S., Jaidee W., Rujanapun N., Duangyod T., Maneerat T., Phuneerub P., Malee K., Popluechai S., Suthiphasilp V., Puttarak P. (2025). Network pharmacology and metabolomics reveal mathurameha, a Thai traditional Anti-Diabetic formula, enhances glucose metabolism through PI3K-AKT/AMPK/GLUT4 pathway modulation. Sci. Rep..

[45] Bradley L.M., Douglass M.F., Chatterjee D., Akira S., Baaten B.J. (2012). Matrix metalloprotease 9 mediates neutrophil migration into the airways in response to influenza virus-induced toll-like receptor signaling. PLoS Pathog..

[46] Gomez I.G., Tang J., Wilson C.L., Yan W., Heinecke J.W., Harlan J.M., Raines E.W. (2012). Metalloproteinase-mediated Shedding of Integrin beta2 promotes macrophage efflux from inflammatory sites. J. Biol. Chem..

[47] Vaisar T., Kassim S.Y., Gomez I.G., Green P.S., Hargarten S., Gough P.J., Parks W.C., Wilson C.L., Raines E.W., Heinecke J.W. (2009). MMP-9 sheds the beta2 integrin subunit (CD18) from macrophages. Mol. Cell Proteom..

[48] Chavey C., Mari B., Monthouel M.N., Bonnafous S., Anglard P., Van Obberghen E., Tartare-Deckert S. (2003). Matrix metalloproteinases are differentially expressed in adipose tissue during obesity and modulate adipocyte differentiation. J. Biol. Chem..

[49] Bradley J.R. (2008). TNF-mediated inflammatory disease. J. Pathol..

[50] Xu Z., Xu J., Li S., Cui H., Zhang G., Ni X., Wang J. (2022). S-Equol enhances osteoblastic bone formation and prevents bone loss through OPG/RANKL via the PI3K/Akt pathway in streptozotocin-induced diabetic rats. Front. Nutr..

[51] Wu N., Yuan T., Yin Z., Yuan X., Sun J., Wu Z., Zhang Q., Redshaw C., Yang S., Dai X. (2022). Network Pharmacology and Molecular Docking Study of the Chinese Miao Medicine Sidaxue in the Treatment of Rheumatoid Arthritis. Drug Des. Devel Ther..

[52] Stanimirovic J., Radovanovic J., Banjac K., Obradovic M., Essack M., Zafirovic S., Gluvic Z., Gojobori T., Isenovic E.R. (2022). Role of C-Reactive Protein in Diabetic Inflammation. Mediat. Inflamm..

[53] Wajant H., Pfizenmaier K., Scheurich P. (2003). Tumor necrosis factor signaling. Cell Death Differ..

[54] Feng X. (2005). RANKing intracellular signaling in osteoclasts. IUBMB Life.

[55] Alipourfard I., Datukishvili N., Mikeladze D. (2019). TNF-alpha Downregulation Modifies Insulin Receptor Substrate 1 (IRS-1) in Metabolic Signaling of Diabetic Insulin-Resistant Hepatocytes. Mediat. Inflamm..

[56] Woo J.R., Bae S.H., Wales T.E., Engen J.R., Lee J., Jang H., Park S. (2024). The serine phosphorylations in the IRS-1 PIR domain abrogate IRS-1 and IR interaction. Proc. Natl. Acad. Sci. USA.

[57] Taniguchi C.M., Emanuelli B., Kahn C.R. (2006). Critical nodes in signalling pathways: Insights into insulin action. Nat. Rev. Mol. Cell Biol..

[58] Molinaro A., Becattini B., Mazzoli A., Bleve A., Radici L., Maxvall I., Sopasakis V.R., Molinaro A., Backhed F., Solinas G. (2019). Insulin-Driven PI3K-AKT Signaling in the Hepatocyte Is Mediated by Redundant PI3Kalpha and PI3Kbeta Activities and Is Promoted by RAS. Cell Metab..

[59] Cho H., Mu J., Kim J.K., Thorvaldsen J.L., Chu Q., Crenshaw E.B., Kaestner K.H., Bartolomei M.S., Shulman G.I., Birnbaum M.J. (2001). Insulin resistance and a diabetes mellitus-like syndrome in mice lacking the protein kinase Akt2 (PKB beta). Science.

[60] Bala A., Roy S., Das D., Marturi V., Mondal C., Patra S., Haldar P.K., Samajdar G. (2022). Role of Glycogen Synthase Kinase-3 in the Etiology of Type 2 Diabetes Mellitus: A Review. Curr. Diabetes Rev..

[61] Force T., Woodgett J.R. (2009). Unique and overlapping functions of GSK-3 isoforms in cell differentiation and proliferation and cardiovascular development. J. Biol. Chem..

[62] Sun B., Chen H., Xue J., Li P., Fu X. (2023). The role of GLUT2 in glucose metabolism in multiple organs and tissues. Mol. Biol. Rep..

[63] Zhang B., Lai G., Wu J., Sun R., Xu R., Yang X., Qi Y., Zhao Y. (2016). 20-HETE attenuates the response of glucose-stimulated insulin secretion through the AKT/GSK-3beta/Glut2 pathway. Endocrine.

[64] Liang G., Wang F., Song X., Zhang L., Qian Z., Jiang G. (2016). 3-Deoxyglucosone induces insulin resistance by impairing insulin signaling in HepG2 cells. Mol. Med. Rep..

[65] Leto D., Saltiel A.R. (2012). Regulation of glucose transport by insulin: Traffic control of GLUT4. Nat. Rev. Mol. Cell Biol..

[66] Wang K., Chen D., Yu B., He J., Mao X., Huang Z., Yan H., Wu A., Luo Y., Zheng P. (2022). Eugenol alleviates transmissible gastroenteritis virus-induced intestinal epithelial injury by regulating NF-kappaB signaling pathway. Front. Immunol..

[67] Beurel E., Grieco S.F., Jope R.S. (2015). Glycogen synthase kinase-3 (GSK3): Regulation, actions, and diseases. Pharmacol. Ther..

[68] Llorens-Martin M., Jurado J., Hernandez F., Avila J. (2014). GSK-3beta, a pivotal kinase in Alzheimer disease. Front. Mol. Neurosci..

[69] Milanski M., Arruda A.P., Coope A., Ignacio-Souza L.M., Nunez C.E., Roman E.A., Romanatto T., Pascoal L.B., Caricilli A.M., Torsoni M.A. (2012). Inhibition of hypothalamic inflammation reverses diet-induced insulin resistance in the liver. Diabetes.

[70] Asimakidou E., Saipuljumri E.N., Lo C.H., Zeng J. (2025). Role of metabolic dysfunction and inflammation along the liver-brain axis in animal models with obesity-induced neurodegeneration. Neural Regen. Res..

