# Supplementary material for: 1-Deoxynojirimycin Combined with Theaflavins Targets PTGS2/MMP9 to Exert a Synergistic Hypoglycemic Effect
Source: Nutrients. 2025 Dec 27;18(1):99. doi: 10.3390/nu18010099 (PMC12787591; doi:10.3390/nu18010099)
Supplement: Supplementary file 1 [file nutrients-18-00099-s001.zip › nutrients-4009258-supplementary.pdf]

Supplementary Table S1 Primers used for qPCR analysis.

Gene	Primer sequences (5′ – 3′)
MMP9	Forward: CTGGTCCTGGTGCTCCTGGTG<br>Reverse: CTGCCTGTCGGTGAGATTGGTTC
PTGS2	Forward: GGGTTGCTGGTGGTAGGAATGTTC<br>Reverse: CTGGTATTTCATCTGCCTGCTCTGG
AKT	Forward: CTGATGATGCCAAGGAGATT<br>Reverse: TGGTCAGGAGGAGTGATTGT
GLUT2	Forward: ATCCTGCTTGGTCTATCTG<br>Reverse: TGATGCTTCTTCCCTTTC
GSK 3α	Forward: AAATCGGTGAAGGAACCTATG<br>Reverse: CCAATACATCGTAAAGTCG
GSK 3β	Forward: ACCTGCCCTCTTCAACTTTACC<br>Reverse: GTGAGGAGGGATAAGGATGGTG
β-Actin	Forward: GGGAAATCGTGCGTGACATTA<br>Reverse: TTGCCGATAGTGATGACCTGA

Supplementary Table S2 Two-way ANOVA of α-Glucosidase inhibition assay

	β	*Se*	*t*	*P*
Intercept	1.0	-	-	<0.001
DNJ (20μg/mL)	-0.41	0.02	-21.58	<0.001
TFs (10μg/mL)	-0.43	0.02	-22.45	<0.001
DNJ-TFs (20μg/mL DNJ+10μg/mL TFs)	-0.07	0.03	-2.48	0.038

DNJ, 1-deoxynojirimycin; TFs, theaflavins.

Supplementary Table S3 Two-way ANOVA of the AUC in the IPGTT

	β	*Se*	*t*	*P*
Intercept	1674.8	17.72	94.50	<0.001
DNJ (200mg/kg)	-230.75	25.07	-9.21	<0.001

TFs (100mg/kg)	-195.56	25.07	-7.80	<0.001
DNJ-TFs (200mg/kg DNJ+100mg/kg TFs)	-101.03	35.45	-2.85	0.008

AUC, area under the curve; IPGTT, intraperitoneal glucose tolerance test; DNJ, 1-deoxynojirimycin; TFs, theaflavins.

Supplementary Table S4 NAS activity score.

	Con	Model	Acarbose	DNJ	TFs	A	B
Hepatic ballooning	0	2	0	0	0	0	0
Lobular inflammation (LI)	0	2	0	1	0	1	0
Steatosis (S)	0	2	0	0	1	0	0
NAS scoring	0	6	0	1	1	1	0

Supplementary Table S5 Topological parameters of 36 key targets of DNJ-TFs against type 2 diabetes

Gene	Centrality	Degrees of Freedom
PTGS2	0.16	31
VEGFA	0.10	30
MMP9	0.05	22
DRD2	0.06	18
ACE	0.12	18
GLB1	0.03	15
GRIA2	0.03	15
GLA	0.06	14
REN	0.02	14
CNR1	0.06	14
SI	0.04	13
MGAM	0.05	13
GRIA1	0.01	13

PRKCA	0.05	13
NR3C1	0.03	12
SLC6A4	0.05	12
MMP2	0.01	12
DPP4	0.06	12
MAPT	0.06	12
GABRA1	0.01	12
SLC1A2	0.01	12
KIT	0.01	11
SLC6A3	0.04	11
GBA	0.04	11
PTGS1	0.01	10
MMP3	0.01	10
MME	0.01	10
AR	0.03	10
MAPK14	0.02	10
LCT	0.02	10
ABCB1	0.05	9
BCHE	0.06	9
GABRB2	0.01	9
MAN2B1	0.01	9
GRIK1	0.03	9
STAT1	0.01	9

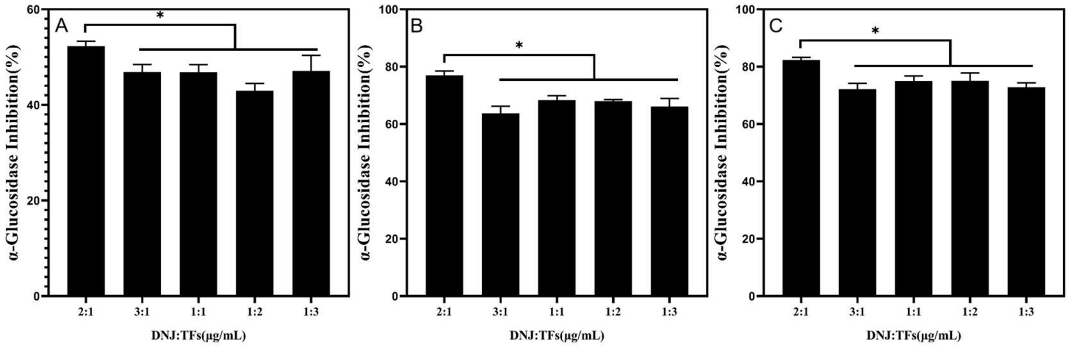

Supplementary Figure S1 α-Glucosidase inhibition rate of DNJ-TFs at different concentration ratios. (A) The α-glucosidase inhibition rate of DNJ-TFs at a final concentration of 5 μg/mL; (B) The α-glucosidase inhibition rate of each proportion at a final concentration of 10 μg/mL; (C) Inhibition rate of α-glucosidase at a final concentration of 20 μg/mL. DNJ, 1-deoxynojirimycin; TFs, Theaflavins. * $P < 0.05$.

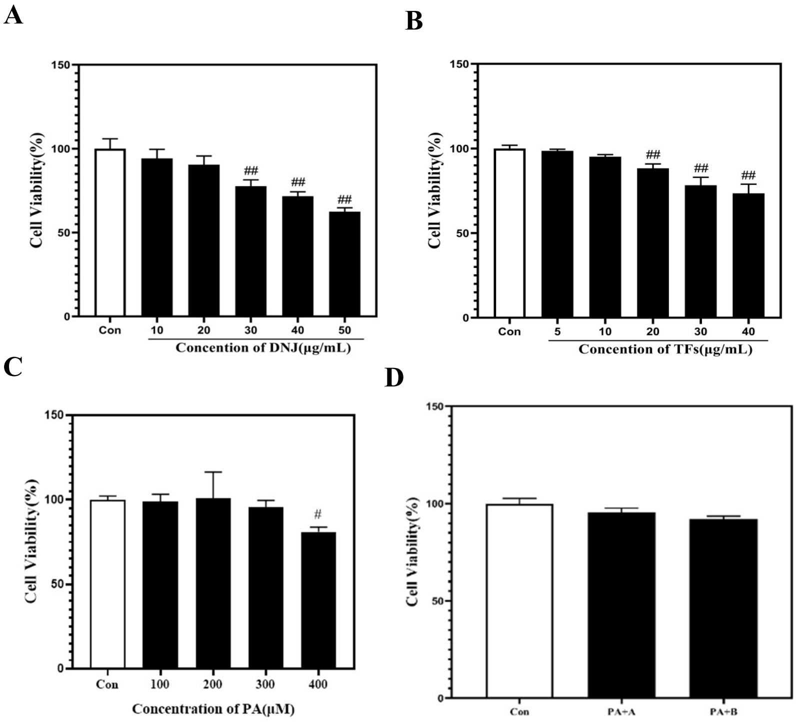

Supplementary Figure S2 Effects of DNJ, TFs and PA on HepG2 cell proliferation detected by CCK-8 assay. (A) Effects of different concentrations of DNJ on the proliferation of HepG2 cells; (B) Effects of different concentrations of TFs on the proliferation of HepG2 cells;(C) Effects of different concentrations of PA on the proliferation of HepG2 cells; (D) Effects of intervention group A and intervention group B on HepG2 cell proliferation, group A: 10 μg/mL DNJ+5 μg/mL TFs; group B: 20 μg/mL DNJ+10 μg/mL TFs. DNJ, 1-deoxynojirimycin; TFs, theaflavins; PA, palmitic acid. # $P < 0.05$, ## $P < 0.01$, versus Con group.

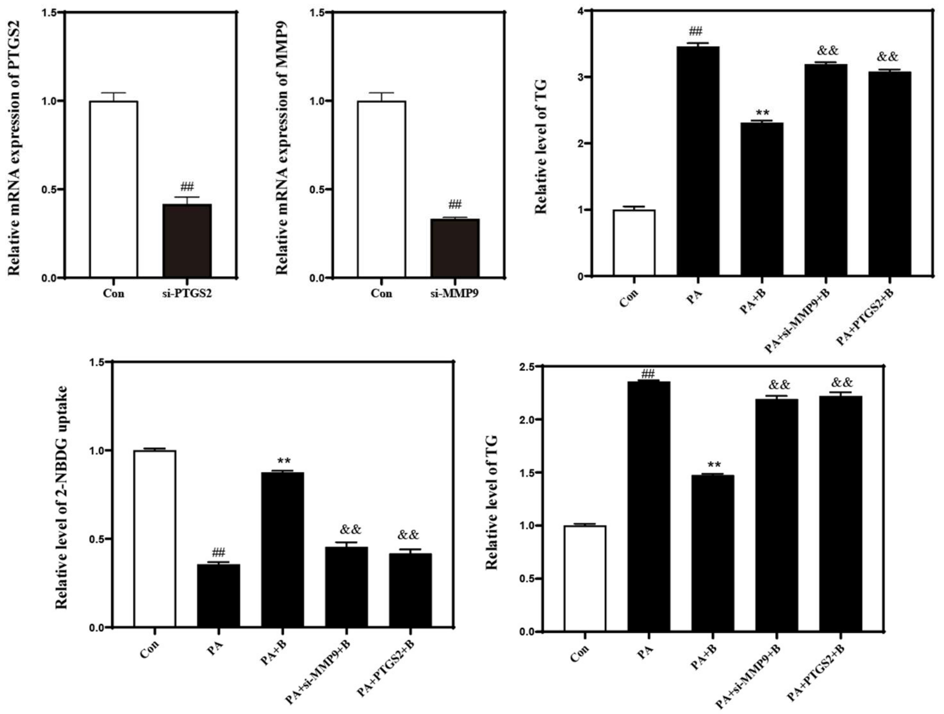

Supplementary Figure S3 The role of DNJ-TFs in hepatocytes after overexpression of PTGS2 and inhibition of MMP9. B: 20 μg/mL DNJ+10 μg/mL TFs for 24 hours. ** $p < 0.01$ vs. PA; ## $p<0.01$ vs. Con; && p < 0.01 vs. PA+B.

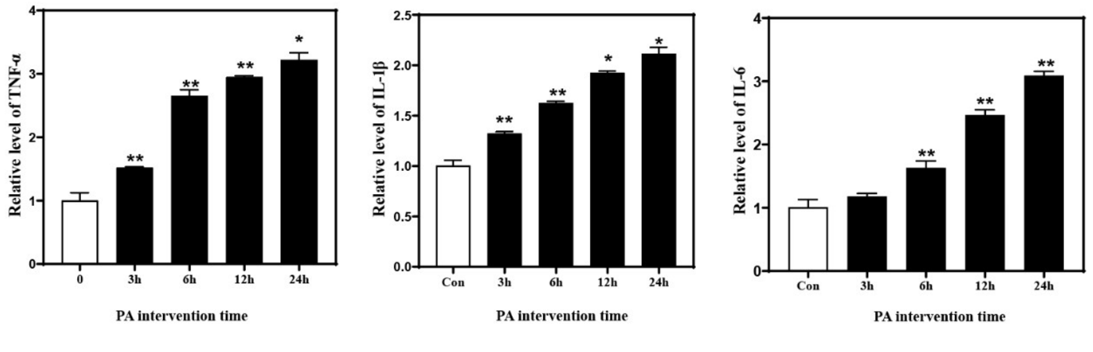

Supplementary Figure S4 Dynamic changes of inflammatory factors at different PA intervention times. PA, palmitic acid. * $P < 0.05$ and ** $P < 0.01$ versus Previous time point.

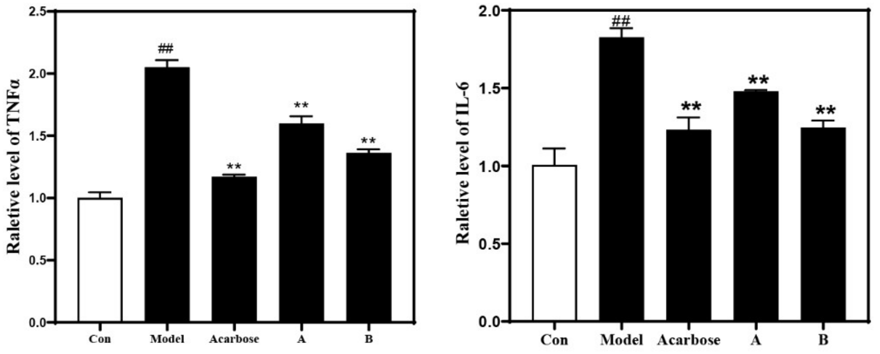

Supplementary Figure S5 Changes in inflammatory factors in liver tissue. A: high-fat+100mg/kg DNJ+50mg/kg TFs; B: high-fat+200 mg/kg DNJ+100 mg/kg TFs. ## $P < 0.01$ versus Con group.** $P < 0.01$ vs Model group.

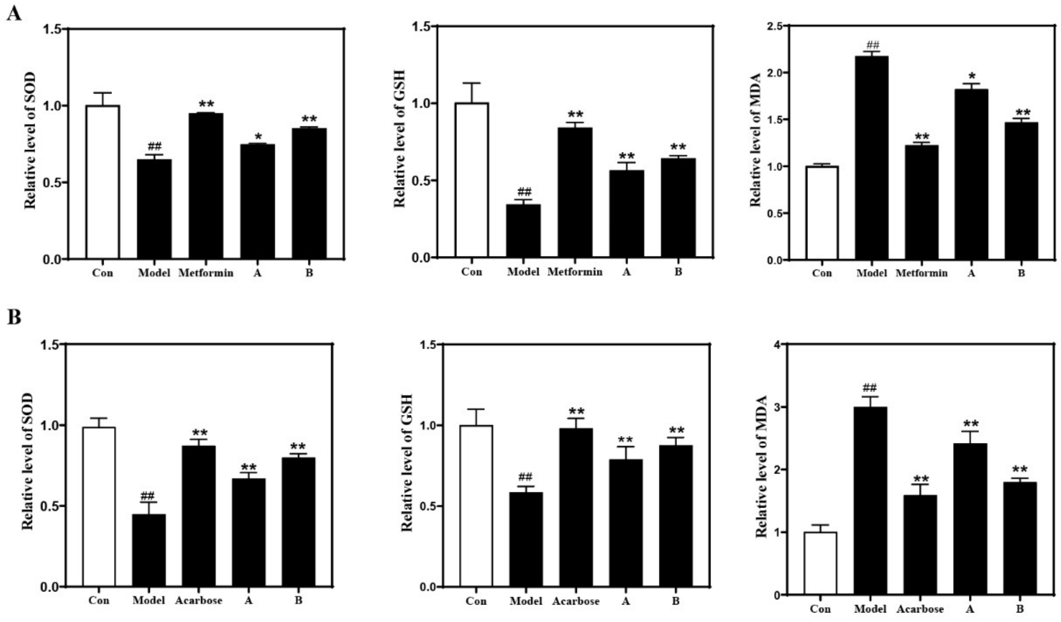

Supplementary Figure S6 Expression levels of oxidative stress factors in mice and HepG2 cells in each group. Expression of inflammatory factors SOD, GSH and MDA in HepG2 cells (A) and mice (B) in each group. SOD, Superoxide Dismutase; GSH, Glutathione; MDA, malondialdehyde. ## $P < 0.01$ versus Con group. * $P < 0.05$ and ** $P < 0.01$ vs Model/Acarbose group.
